# Hearing aids to support cognitive functions of older adults at risk of dementia: the HearCog trial- clinical protocols

**DOI:** 10.1186/s12877-020-01912-1

**Published:** 2020-11-26

**Authors:** Dona M. P. Jayakody, Osvaldo P. Almeida, Andrew H. Ford, Marcus D. Atlas, Nicola T. Lautenschlager, Peter L. Friedland, Suzanne Robinson, Marshall Makate, Lize Coetzee, Angela S. P. Liew, Leon Flicker

**Affiliations:** 1 Ear Science Institute Australia, 1 Salvado Road, Subiaco, WA 6008 Australia; 2 Ear Sciences Centre, Faculty of Health and Medical Sciences, University of Western Australia, 35 Stirling Highway, Perth, WA 6009 Australia; 3 Western Australian Centre for Health and Ageing, Medical School, Faculty of Health and Medical Sciences, University of Western Australia, 35 Stirling Highway, Perth, WA 6009 Australia; 4 Academic Unit for Psychiatry of Old Age, Department of Psychiatry, The University of Melbourne, Parkville, VIC 3010 Australia; 5 North Western Mental Health, Melbourne Health, Parkville, VIC 3010 Australia; 6 Department of Otolaryngology, Head Neck Skull Base Surgery, Sir Charles Gairdner Hospital, Hospital Ave, Nedlands, WA 6009 Australia; 7 School of Medicine, University Notre Dame, Fremantle, WA 6160 Australia; 8 Curtin University, School of Public Health, Kent St, Bentley, WA 6102 Australia

**Keywords:** Hearing loss, Hearing aids, Dementia, Depression, Frailty, Cognition, Cognitive decline

## Abstract

**Background:**

Globally, about 50 million people were living with dementia in 2015, with this number projected to triple by 2050. With no cure or effective treatment currently insight, it is vital that factors are identified which will help prevent or delay both age-related and pathological cognitive decline and dementia. Observational data have suggested that hearing loss is a potentially modifiable risk factor for dementia, but no conclusive evidence from randomised controlled trials is currently available.

**Methods:**

The HearCog trial is a 24-month, randomised, controlled clinical trial aimed at determining whether a hearing loss intervention can delay or arrest the cognitive decline. We will randomise 180 older adults with hearing loss and mild cognitive impairment to a hearing aid or control group to determine if the fitting of hearing aids decreases the 12-month rate of cognitive decline compared with the control group. In addition, we will also determine if the expected clinical gains achieved after 12 months can be sustained over an additional 12 months and if losses experienced through the non-correction of hearing loss can be reversed with the fitting of hearing aids after 12 months.

**Discussion:**

The trial will also explore the cost-effectiveness of the intervention compared to the control arm and the impact of hearing aids on anxiety, depression, physical health and quality of life. The results of this trial will clarify whether the systematic correction of hearing loss benefits cognition in older adults at risk of cognitive decline. We anticipate that our findings will have implications for clinical practice and health policy development.

**Trial registration:**

Australian and New Zealand Clinical Trials Registry (ANZCTR: 12618001278224), registered on 30.07.2018.

## Background

Hearing loss is the second highest cause of disability in the world, affecting 466 million people, with 90% of cases being due to age-related hearing loss (ARHL) [1]. ARHL is a highly prevalent form of sensory impairment in later life, affecting 40 to 45% of people aged 65 years and 83% of those aged 70 years or above [2]. ARHL increases the risk of mental health problems [3], frailty [4], cognitive impairment [5] and dementia [6].

Currently, more than 50 million people are living with dementia, and this is projected to reach 75.63 million in 2030 and 135.46 million in 2050 [7]. According to the Lancet Dementia Taskforce, of the many risk factors that contribute to dementia, hearing loss could account for 8% of all dementia cases [8]. Developing effective strategies to prevent dementia has become a global health priority, with projections suggesting that the total number of people living with dementia could be reduced by 13% if the onset of symptoms could be delayed by two years or more [9].

Australian data from the Health In Men Study showed that in a sample of 37,898 older men, the hazard of dementia associated with hearing impairment was 1.69 (95%CI = 1.54, 1.85) [10]. In addition, a systematic review of 14 prospective studies showed that hearing loss was associated with a 49% (95%CI 30–67%) increase in the hazard of dementia [10]. Whilst this data shows a clear association between hearing loss and cognitive impairment, a causal relationship cannot be definitively determined, and it is currently not known whether the correction of hearing loss through the use of hearing aids can decrease the rate of cognitive decline or reduce dementia risk.

Our trial aims to investigate whether the correction of hearing loss through the use of hearing aids (HAs) could decrease the 12-month rate of cognitive decline among older adults at risk of dementia.

## Methods

### Aims

1. This trial will determine whether the correction of hearing loss through the use of hearing aids (HA) decreases the 12-month rate of cognitive decline among older adults at risk of dementia.
2. We will also investigate whether the correction of hearing loss has a beneficial impact on memory and executive functions, anxiety and depressive symptoms, quality of life, physical health, and health-related costs over 12 months.
3. We will explore whether the expected clinical gains achieved through the correction of hearing loss by 12 months can be sustained over an additional period of 12 months and if losses experienced through the non-correction of hearing loss can be reversed with the fitting of HAs after 12 months (i.e., HAs fitting for controls at 12 months with follow up of 12 months).

### Study design

Two-arm parallel randomised controlled trial.

### Participants and setting

We will recruit 180 older adults with mild cognitive impairment and hearing loss. The trial will be conducted at the Ear Science Institute Australia (ESIA) based in the Perth and Bunbury metropolitan regions, Western Australia as well as the Western Australian Centre for Health & Ageing, Perth, Australia. Participants will be recruited from the ESIA hearing clinics, aged-care homes, and hospital memory clinics. In addition, we will place advertisements in the local media and primary care networks, inviting interested participants for screening. If the recruitment of participants is lower than predicted, we will use the electoral roll list to select a random list of people aged ≥70 years living the study areas: they will receive information about the study and an invitation to contact the research office for screening if they believe they may be potentially eligible (the mail out will be de-identified – i.e., investigators will not have access to the list). This approach has been used successfully in other studies.

### Eligibility criteria

Participants will:

- Be older adults aged 70 years or older (cognitive decline is more pronounced later in life).
- Have a Montreal Cognitive Assessment for the Hearing Impaired (HI-MOCA [11] greater than 18 and less than 26 (mild impairment).
- Have better ear average hearing loss at 0.5, 1 & 2 kHz (3FAHL) > 23 dB or high-frequency average hearing loss (2, 3 & 4 kHz) (HFAHL) ≥ 40 dB as measured using air conduction pure-tone audiometry (HA fitting criteria recommended by Hearing Services Program in Australia for older adults with ARHL [12].
- Be fluent English speakers.

### Exclusion criteria

We will exclude participants who:

- Have impaired instrumental activities of daily living (IADL) [13] due to cognitive deficits (requires assistance or is dependent in the use of telephone, shopping, housekeeping, laundry, transport, management of medications and finances) – i.e. have dementia [14] or major neurocognitive disorder.
- Meet clinical criteria for cochlear implantation (unaided bilateral sensorineural hearing loss > 70 dBHL, and open-set sentence scores in quiet in the worse ear < 65% and in the better ear < 85% or open set phoneme scores in quiet in the worse ear < 45% and in the better ear < 65% with optimised HA fitting [15].
- Have visual impairment that limits participant’s ability to read Times New Roman font size 16 (a requirement for two sentences of HI-MOCA).
- Have a severe medical illness that limits the ability of the participant to attend appointments or sustain participation in the study for 24 months.
- Plan to move away from the study area during the subsequent 24 months.
- Are unable or unwilling to provide written, informed consent to participate.
- Are unable to complete the motor screening task (MOT) module of the Cambridge Neuropsychological Test Battery (CANTAB) due to visual impairment, inability to comprehend test instructions or inability to attend to the task due to dexterity problems [16].

### Intervention

The intervention consists of three parts: (i) hearing assessment and HA discussion, (ii) HA fitting, verification and validation and (iii) HA review following daily use of HAs.

The intervention will be carried out by a qualified audiologist according to the Australian Audiological Society Standards in a standardised soundproof booth. All participants will be given a pair of Oticon OPN 1S hearing aids.

### Outcomes

We will use several well-validated scales to assess our primary and secondary outcomes.

#### Primary outcome measures

*Global cognitive abilities:* Due to hearing impairment, older people may experience difficulty in following verbal instructions or completing tasks that heavily rely on hearing during cognitive assessments. This may result in overestimation of cognitive impairment in such individuals [5]. Hence, we have used a non-verbal global cognitive measure that has been validated to use with the hearing impaired older adults. The global cognitive abilities will be measured using the Montreal Cognitive Assessment for the Hearing Impaired (HI- MoCA [11]. No significant difference was observed for MOCA and HI-MOCA scores in cognitively intact normal hearing participants, and the test-retest reliability coefficient was 0.66 [11].

#### Secondary outcome measures

*Nonverbal cognition assessment using Cambridge Neuropsychological Test Battery (CANTAB)* [16]*- This assessment does NOT rely on verbal communication:*

- Attention Switching task (AST): is a test of executive functioning and provides a measure of cued attentional set-shifting [16]. AST is based on the Stroop test and relies heavily on the functions of the anterior right hemisphere and medial frontal structures.
- Delayed Matching Sample (DMS): assesses participants’ ability to recognise complex visual patterns at different time intervals [16]. It is primarily sensitive to medial temporal lobe dysfunction.
- Paired Associates Learning (PAL): PAL is a recall test of memory which assesses episodic visuospatial memory, learning and association ability [16]. PAL is primarily sensitive to the changes in medial temporal lobe functioning.
- Spatial Working Memory (SWM): measures the retention and manipulation of visuospatial information in areas such as non-verbal working memory, working visuospatial memory and strategy use [16].

### Other measures including safety measures

#### General physical & mental health

Participants will be asked to complete the following widely used and validated assessments:

- Cognitive reserve questionnaire to obtain information on participant age, gender, education, work history and leisure activities [17].
- Health status and Quality of life: Short Form survey (SF-12) [18].
- Physical function: Functional Comorbidity Index (FCI) [19].
- Depressive symptoms: Patient Health Questionnaire (PHQ-9) [20].
- Anxiety symptoms: Geriatric Anxiety Inventory (GAI) [21].
- Function: Lawton & Brody Instrumental Activities of Daily Living (IADL) [22].
- Social Support and interaction: de Jong Gierveld social support questionnaire [23]^.^
- Frailty: handgrip strength will be measured using a Jamar Analogue Hand Dynamometer [24].
- Psychological and social adjustment problems resulting from hearing loss: Hearing Handicap Inventory of the Elderly (HHIE) [25].
- Effectiveness of the HAs application: International Outcome Inventory for HAs (IOI-HA) [26].
- Demographic questionnaire
- Health-care utilisation cost questionnaire.

#### Hearing Assessment

The assessment of hearing will consist of two parts:

- Peripheral hearing assessment will be based on tympanometry, which provides information about middle ear pathologies; pure-tone audiometry, which generates information on hearing thresholds across .25–.8 kHz frequency range; and speech perception in a quiet environment: Consonant-Nucleus-Consonant (CNC) word [27] and City University of New York (CUNY) sentence test [28].
- Central hearing assessment will comprise of the following tests: Dichotic Digits Test (DDT) [29], Synthetic Sentence Identification with Ipsilateral Competing Message (SSI-ICM) [30] and Quick Speech in Noise (Quick-SIN) [31].

### Procedures for the collection of study measures

**Fig. 1:**
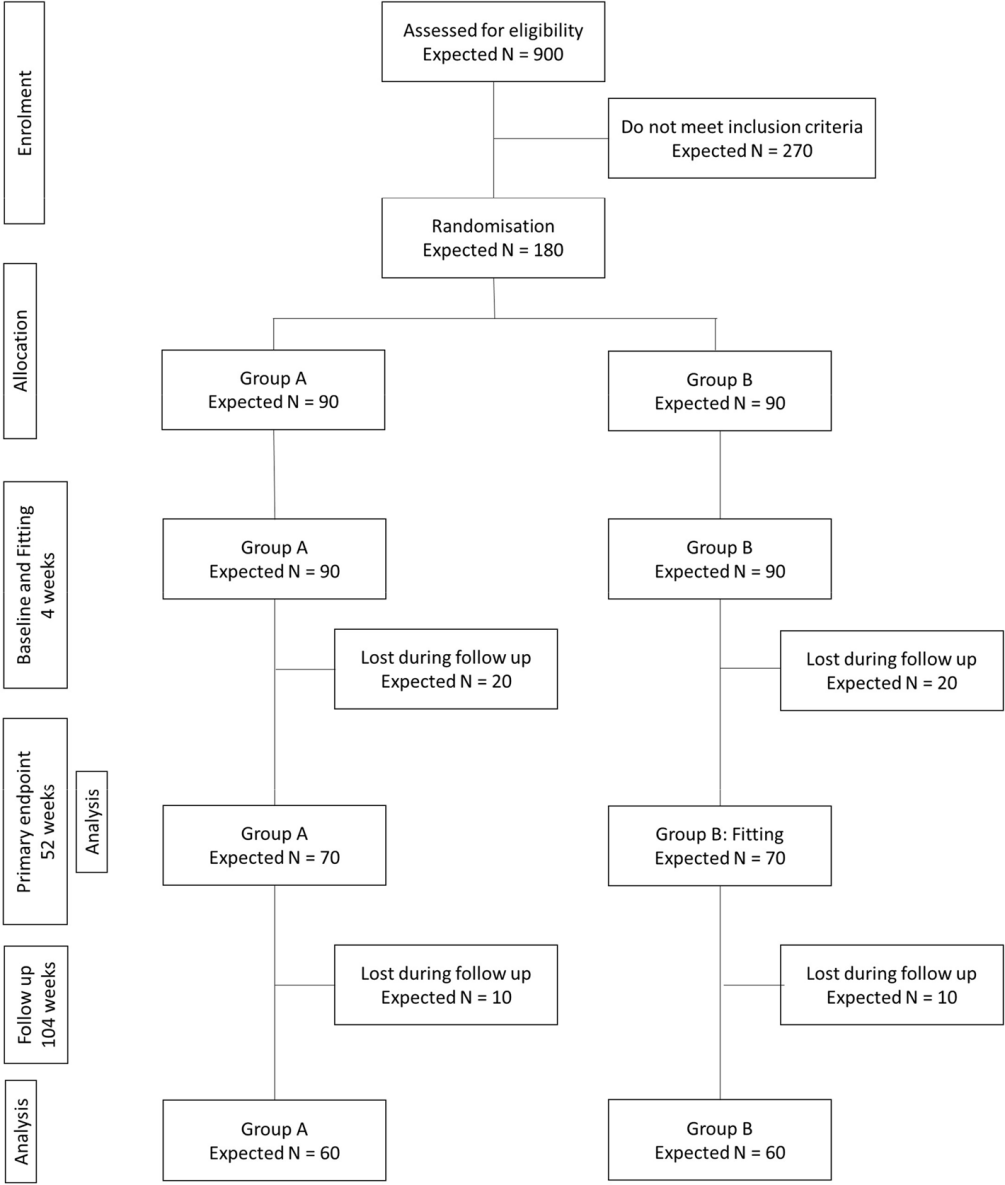
Anticipated participant recruitment flowchart

The procedure for the data collection will follow the Consolidated Standards of Reporting Trials (CONSORT) guidelines. During the screening process, participants who meet the criteria for inclusion in the study will be randomly assigned to either the experimental (A) or control (B) group. Group A participants will receive the intervention immediately after the baseline assessment, whereas group B participants will receive the intervention 12 months later (Fig. 1). All participants will be informed that if they get randomly allocated to group B, they will have to wait 12 months to receive the treatment. Those who prefer to receive HAs immediately without having to wait 12 months will be given the option to opt-out from the study. Cognition, mental health and QoL assessments will be carried out separately to the hearing assessments and HA fitting.

Group A will complete hearing assessment, cognitive, mental health and QoL assessment at the baseline, 6, 12, 18 and 24 months.

Group B will complete hearing assessment, cognitive, mental health and QoL assessment at the baseline, 6, 12 months, 18 and 24 months. (Primary endpoint analysis at 12 months and follow-up analysis at 24 months are shown in Fig. 1). Timeline of the study is shown in Table 1.

**Table 1:**
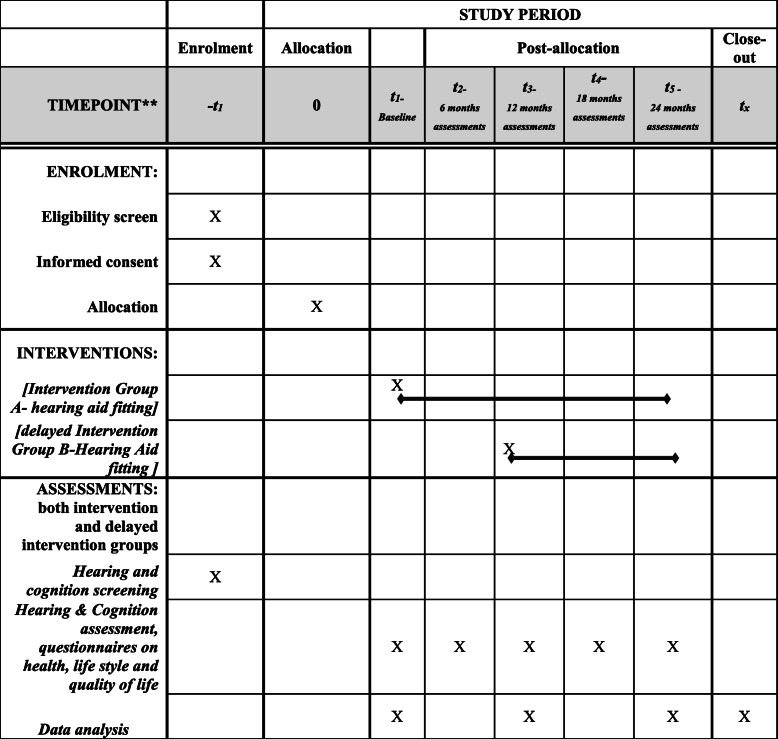
Timeline of the study

**Intervention:** will be conducted by a qualified Audiologist.

**Part I: Hearing assessment and HA discussion**

During this appointment, the participant will complete (1) a comprehensive case history on their medical and hearing history, (2) a Client Oriented Scale of Improvement (COSI) goals [32] for everyday listening situations and (3) a standard hearing assessment. An explanation on what are hearing aids and how they work, what they are used for, how to use them, and related questions and answers will be provided.

**Part II:** HA fitting, real-ear verification and validation -immediately following appointment part I.

The audiologist will program the HA and carry out the real-ear verification using real-ear insertion gain (REIG) to ensure that appropriate amplification is provided validation tasks will be carried out to determine that the participant is benefitting from the HAs [33]. Adjustments will be made to the devices so that the participant is comfortable with the devices.

**Part III: HA review:** 2 weeks after the HA fitting.

HA data logging information recorded in the software of the HA is analysed to ensure that the HA program provides the best solutions to the listening demands of the participant. Based on COSI goals, data logging information and feedback received from the participants, changes are made to the HA program.

### HA review appointments at 12 and 24 months after HA fitting

These appointments are similar to Part II and III of the HA fitting appointments. During these appointments, a standard pure-tone audiometric assessment to obtain hearing thresholds, reprogramming of the HA according to the current hearing loss.

### Measuring adherence with treatment

The Oticon Opn 1S HAs have a “log in“feature that records both the average number of hours and different listening environments in which the participant has used the HA. These data can be retrieved when the HA is connected to the program software, which will be done at all assessments. In addition, the participant will be asked to maintain a daily listening diary in which s/he records the number of hours the HA is worn.

### Pilot test and sample size

In preparation of this trial, we conducted a pilot observational intervention study of two groups of hearing-impaired older adults: Group 1 [*n* = 35, mean age = 70.2 + 6.7 years, better ear four frequency average .5, 1, 2 & 4 kHz (BE 4PTA) = 31.92 dB, better ear high frequency average of 6 & 8 kHz (BE HF2PA) = 54.07 dB] and Group 2 [*n* = 13, mean age = 71.8 + 7.4 years, BE 4PTA = 33.46 dB, BE 2HFPTA = 55.57 dB]. A control group of 19 normal-hearing participants was also included. All participants completed hearing and a non-verbal cognitive assessment using the CANTAB test battery at baseline, 6 and 12 months. Hearing aids (HA) were fitted to Group 2 participants after the baseline assessment. Analysis of variance revealed that Group 2 participants (HA users) performed significantly better than Group 1 (non-HA users) on the delayed matching-to-sample (DMS) test of the CANTAB battery (*p* = .02, d = 0.38). We used G*Power software [34] to determine the required sample size for the study. Based on these pilot data, we calculated that a total of 140 participants would be required (70 in each group) to detect a conservative effect size of the intervention of d = 0.27 with two-sided α set at .05 and power of .90. To account for 25% of attrition over time, we estimated that a total of 180 participants would need to be recruited.

### Randomisation, concealment and blinding

The computer-generated randomisation sequence will be stratified by the severity of the hearing loss (mild to moderate vs severe) based on the results of the hearing assessment. Each stratification block will be associated with a random sequence of numbers assigned to the intervention and control group in random permuted blocks of 6, 8 or 10. This sequence will be stored in a password-protected server housed at the University of Western Australia and will be managed by a biostatistician not involved in this project. Once a participant consents and is enrolled, s/he will be automatically ascribed a number and group membership (intervention or control).

Due to the nature of the intervention, participants will know their group assignment, but research staff involved in the assessment of cognitive function, quality of life, mood and physical function will remain blind to treatment allocation. This will be achieved by directing participants to **NOT**: (i) discuss any aspects of the intervention during the assessments, (ii) wear their HAs during the assessment. Binaural hearing amplifiers will be used to facilitate the communication between participants and research staff during all assessment visits (including the 12 and 24-month visits).

### Health economic analysis

This will involve the development of a model to estimate the incremental cost-effectiveness of the intervention compared to the control. The analyses will be from the perspective of the health service and will be expressed as Quality-Adjusted Life Years gained. A particular focus of the economic evaluation will be a full assessment of the cost of delivering the intervention compared to that of the control group (including the costs of intervention material, costs of procedures, visits to health service provides and list of all medications). Given the feasibility of obtaining health administrative data within the study time frame, we will use a validated patient cost questionnaire to obtain self-reported health care utilisation data [35]. Whilst we recognise the potential for recall bias, there is evidence to suggest that this is a valid method of collecting data on health-care resource utilisation for use in economic evaluations, especially when administrative data is not easily available [36]. Costings information will be applied based on established economic costing methodologies drawing on primary research and secondary national tariffs [37]. Further, an application will be made to the Department of Health Linked data systems obtain pharmaceutical-based costs, Medicare-based costs and other associated costs including Mental Health Information System, Home and Community Care, Emergency Department Data, Aged Care Assessment and St John Ambulance related to each participant of the study.

The second aspect will include the assessment of the effectiveness of the intervention – effectiveness of the intervention and control will be measured using the SF-12, which is widely used in economic evaluations.

Incremental cost-effectiveness ratios will be calculated in terms of the incremental cost per sustained remission and the incremental cost per Quality-Adjusted Life Year (QALY) gained by the intervention. The QALY is a widely-used approach for estimating the quality of life benefits in economic evaluations. The values obtained from the SF-12 will be transformed into utility weights using the Short Form 6D algorithm [38] to formulate the cost per QALY. Sensitivity analysis will be undertaken to test the robustness of results.

### Statistical analysis

All analyses will follow CONSORT guidelines. We will use standard descriptive statistics to compare basic sociodemographic and clinical data across treatment arms. We will use multilevel mixed models to investigate changes in cognitive and other scale scores over time. Mixed models provide estimates that are “intention-to-treat’ and allow for the investigation of interactions between group and time effects, as well as for the adjustment of possible imbalances between the groups following the randomisation. We will use imputed chain equations if the loss to follow up exceeds 25%. All probability tests will be two-tailed.

## Discussion

The current paper discusses the methodology for a randomised control trial that investigates whether hearing loss intervention using hearing aids could delay or arrest the cognitive decline in older adults with mild cognitive impairment. One of the strengths of this trial is that it follows CONSORT guidelines for the design of randomised controlled trials. The recruitment of participants with mild cognitive deficits was guided by our desire to test a population at risk of dementia (when prevention may be possible) and by the difficulties associated with the consenting of older adults with moderate to severe cognitive impairment. Besides, those with severe to profound hearing loss who meet the criteria for a cochlear implant will not benefit from HA amplification, hence, including them would potentially undermine the impact of HA amplification on cognitive functions, mental health and QoL. We acknowledge, however, that our study will focus on the cognitive decline rather than conversion to dementia. At this stage, this is a “proof of concept’ investigation, as a dementia prevention trial would require a substantially larger sample and follow up. The projected outcomes of the current study can immediately be translated to practice through audiology clinics and will be applicable across practices around the world. Findings can also be used to inform audiologists, general practitioners and other health-care providers. This will provide important information for older people about the use of hearing aids to prevent worsening cognitive impairment. In addition, consumer support will be requested in disseminating lay summaries/information to the community. If cognitive decline can be delayed or arrested, this would improve the quality of life of older adults who are at risk of developing dementia. It may also lower costs to the health-care and social support systems, by decreasing the needs for services and residential care placement. It would also significantly reduce the overall burden borne by the community.

## Data Availability

Not applicable.

## Abbreviations

ARHL: Age-related hearing loss
HA: Hearing aids
ESIA: Ear Science Institute Australia
HI-MOCA: Montreal Cognitive Assessment for the Hearing Impaired
3FAHL: Three Frequency Average Hearing Loss
HFAHL: High Frequency Average Hearing Loss
IADL: Instrumental Activities of Daily Living
dBHL: Decibel Hearing Loss
MOT: Motor Screening Task
CANTAB: Cambridge Neuropsychological Test Battery
AST: Attention Switching task
DMS: Delayed Matching Sample
PAL: Paired Associates Learning
SWM: Spatial Working Memory
SF-12: Health status and Quality of life: Short Form Survey
FCI: Functional Comorbidity Index
PHQ-9: Patient Health Questionnaire
GAI: Geriatric Anxiety Inventory
HHIE: Hearing Handicap Inventory of the Elderly
IOI-HA: International Outcome Inventory for HAs
CNC: Consonant-Nucleus-Consonant
CUNY: City University of New York
DDT: Dichotic Digits Test
SSI-ICM: Synthetic Sentence Identification with Ipsilateral Competing Message
Quick-SIN: Quick Speech in Noise
QoL: Quality of Life
COSI: Client Oriented Scale of Improvement
REIG: Real Ear Insertion Gain
CONSORT: Consolidated Standards of Reporting Trials
QALY: Quality Adjusted Life Year
